# Organ-Specific Metabolome Deciphering Cell Pathways to Cope with Mercury in Wild Fish (Golden Grey Mullet *Chelon auratus*)

**DOI:** 10.3390/ani12010079

**Published:** 2021-12-30

**Authors:** Giuseppe De Marco, Fátima Brandão, Patrícia Pereira, Mário Pacheco, Tiziana Cappello

**Affiliations:** 1 Department of Chemical, Biological, Pharmaceutical and Environmental Sciences, University of Messina, 98166 Messina, Italy; gdemarco@unime.it; 2 Centre for Environmental and Marine Studies (CESAM), Department of Biology, University of Aveiro, Campus Universitário de Santiago, 3810-193 Aveiro, Portugal; fatimabrandao.988@gmail.com (F.B.); pkpereira@ua.pt (P.P.); mpacheco@ua.pt (M.P.)

**Keywords:** fish metabolome, NMR-based metabolomics, *Chelon auratus*, liver, gills, polar metabolites, mercury pollution

## Abstract

**Simple Summary:**

Metabolomics is a powerful approach that is based on the identification in biological samples of metabolites, which production and levels may vary due to factors intrinsic to the environment and the organism. For a correct data interpretation, it is, therefore, necessary to first evaluate the metabolome of the tissue/organ under investigation when it is exposed to no stressor. In this study, the complete set of metabolites of liver and gills of wild golden grey mullet (*Chelon auratus*) that were collected from a reference area was compared by using metabolomics, which was able to reveal metabolites that are commonly present in both organs but with different levels to be attributed to organ-specific functions. The same metabolomic approach was applied also to study the metabolite changes that were induced in mullet gills and liver after environmental exposure to mercury (Hg), and a variety of organ-specific metabolic disturbances were observed. The findings from this study validate the use of metabolomics in ecotoxicological studies to assess organ-specific functions and the cytotoxicity mechanisms of Hg in fish.

**Abstract:**

Metabolomics is a powerful approach in evaluating the health status of organisms in ecotoxicological studies. However, metabolomics data reflect metabolic variations that are attributable to factors intrinsic to the environment and organism, and it is thus crucial to accurately evaluate the metabolome of the tissue/organ examined when it is exposed to no stressor. The metabolomes of the liver and gills of wild golden grey mullet (*Chelon auratus*) from a reference area were analyzed and compared by proton nuclear magnetic resonance (^1^H NMR)-based metabolomics. Both organs were characterized by amino acids, carbohydrates, osmolytes, nucleosides and their derivatives, and miscellaneous metabolites. However, similarities and differences were revealed in their metabolite profile and related to organ-specific functions. Taurine was predominant in both organs due to its involvement in osmoregulation in gills, and detoxification and antioxidant protective processes in liver. Environmental exposure to mercury (Hg) triggered multiple and often differential metabolic alterations in fish organs. Disturbances in ion-osmoregulatory processes were highlighted in the gills, whereas differential impairments between fish organs were pointed out in energy-producing metabolic pathways, protein catabolism, membrane stabilization processes, and antioxidant defense system, reflecting the induction of organ-specific adaptive and defensive strategies. Overall, a strict correlation between metabolites and organ-specific functions of fish gills and liver were discerned in this study, as well as organ-specific cytotoxicity mechanisms of Hg in fish.

## 1. Introduction

Metabolomics is a powerful and sensitive tool to evaluate the health status of aquatic organisms in environmental ecotoxicological studies [1]. It provides complete and accurate information on the biochemical responses to contaminants exposure, complementing evidence that are provided by the use of conventional biomarkers [2,3]. Besides that, metabolomics has been contributing with valuable novel insights on the specific mechanisms of aquatic contaminants toxicity in fish. A paradigmatic example of such usefulness is demonstrated by the constant increase in the number of metabolomic studies dealing with ecotoxicology that have been produced in the last two decades, with about 900 papers on this topic published annually [4,5].

Although mercury (Hg) is widely known for its toxicity in humans and wildlife, the number of studies using metabolomics to address its effects in fish is still scarce. In our previously published papers, we elucidated the mechanisms of environmental Hg-toxicity in wild golden grey mullet (previously named as *Liza aurata*, now *Chelon aurata*) by using an innovative triad approach that was based on Hg bioaccumulation, shifts in metabolite profiles, and conventional oxidative stress biomarkers [2,3,6]. This strategy was successfully applied to mullets inhabiting an Hg-contaminated system in the Aveiro lagoon, Portugal. As a consequence of the high accumulated levels of inorganic Hg (iHg) and methylmercury (MeHg), severe changes were documented in mullet gills in the metabolites that were related to the antioxidant protection, with depletion of reduced glutathione (GSH) and its constituent amino acids (i.e., glutamate and glycine) [3]. This was further corroborated through oxidative stress endpoints, by depletion of glutathione peroxidase (GPx) and superoxide dismutase activities, indicating massive GSH oxidation under Hg stress and an inability to carry out its regeneration or de novo synthesis [3]. Alterations in the structure of cell membranes were also suggested, together with alternative mechanisms for preventing lipid peroxidative damage [3,6]. A mechanistically-based assessment of Hg toxicity was conducted also in liver of the same wild mullets, providing new insights into the toxicological pathways underlying the oxidative stress at hepatic level [2]. Metabolomics revealed several levels of impact due to Hg exposure that triggered adaptive responses in the antioxidant system as observed by increased glutathione-*S*-transferase and catalase activities, and total glutathione content, which compensated for a decreased GPx activity. Despite these effects, the induction of lipid peroxidation was efficiently prevented in mullet liver [2]. Noteworthy, some of the adaptive and defensive strategies that were triggered by Hg contamination were similar in mullet gills and liver, while others reflected organ-specific responses. It is, therefore, of high relevance in ecotoxicological studies to deeply investigate and compare the metabolite profile of mullet gills and liver, and then observe any organ-specific deviations that are triggered by Hg exposure. Both these issues were addressed in the present work, conducted on the same individuals that were used in our previous studies.

Metabolomics was also applied to unveiling the toxicity of Hg by Bridges et al. [7] that sequenced the gut microbiome of fathead minnows (*Pimephales promelas*) that were exposed to dietary MeHg to investigate its relation to neurotoxicity. Data suggested that environmentally relevant exposure scenarios might cause MeHg-mediated dysbiosis of the gut microbiome, contributing to neurotoxicity in fish. Recently, the effects of MeHg and iHg were investigated in zebrafish embryos [8]. The metabolism of galactose, starch, and sucrose was disturbed after exposure to both Hg forms, and the levels of the neurotransmitters tyrosine, dopamine, and tryptophan were reduced. In addition, oxidative stress was related to metabolite changes, such as alterations in the putrescine, niacinamide, and uric acid contents in zebrafish that were exposed to iHg, and squalene in the MeHg-exposed organisms [8].

Fish gills and liver are organs that are widely used in studies addressing environmental biomonitoring of pollutants, including Hg [2,3,9,10]. While the gills are the main organ of waterborne Hg uptake, the liver accumulates high levels of Hg upon dietary exposures due to its main role in metabolism. Indeed, a toxicokinetic study of waterborne iHg in white seabream (*Diplodus sargus*) pointed out the gills as the most responsive organ by accumulating the metal faster than the other surveyed tissues/organs (eye wall, lens, blood, liver, brain, and bile), while accumulating the highest levels over the exposure time [11]. Moreover, a recent comparative study of dietary iHg and MeHg toxicity in the Korean rockfish (*Sebastes schlegeli*) comprised of the evaluation of Hg accumulation in several tissues (liver, kidney, dorsal muscle, and brain), reporting the highest levels in the liver upon exposure to MeHg [12]. This pattern was explained by the primary role of liver in detoxification that is related to metallothioneins or metallothionein-like proteins that bind specific metals (e.g., Hg, cadmium, copper) and sequester Hg ions [13]. Keeping in mind that, under realist exposure scenarios, fish are mainly exposed to waterborne iHg and dietary MeHg [14], the gills and liver should be combinedly considered in fish health assessment to better understand the extent of the effects that are triggered by these two Hg forms.

Data that were obtained from metabolomic investigative studies reflect metabolic variations that are attributable to factors intrinsic to organisms (e.g., age and developmental stage, gender, size, feeding condition, genetic, specificities of tissues targeted, etc.) and to the environment (e.g., temperature, salinity, dissolved oxygen, etc.). Therefore, to avoid the interference of these confounding factors, it is crucial to evaluate first the metabolome of the tissue/organ under investigation when it is exposed to no stressor. Cappello, et al. [15] published the metabolome of farmed mussel (*Mytilus galloprovincialis*) through the investigation of three different organs, namely the digestive gland, gills, and posterior adductor muscle, reporting a total of 44 metabolites that were grouped in amino acids, carbohydrates, tricarboxylic acid (TCA) cycle intermediates, osmolytes, neurotransmitters, nucleotides, alkaloids, and miscellaneous metabolites. Multivariate statistics revealed that mussel organs clustered separately from each other, suggesting a clear differentiation in their metabolic profiles. Therefore, the study of Cappello, et al. [15] provided a better understanding of mussel organ-specific functions, while supporting future metabolomic investigations of marine mussel health and safety. A similar approach would be valuable in fish to better address the toxicity of aquatic contaminants [16].

In the current study, the metabolomes of the gills and liver of the wild golden grey mullet (*Chelon auratus*) were analyzed by a proton nuclear magnetic resonance (^1^H NMR)-based metabolomic approach to achieve: (i) a comparison of both organs of fish that were captured at a putative reference area that was considered unpolluted for Hg to highlight the similarities and differences that are related to organ-specific functions; (ii) a comparison of both organs of mullets that were captured at an Hg-polluted area in respect to those from the reference area to detect any metabolite deviations that were triggered by environmental exposure to Hg.

## 2. Materials and Methods

### 2.1. Study Area

**Figure 1:**
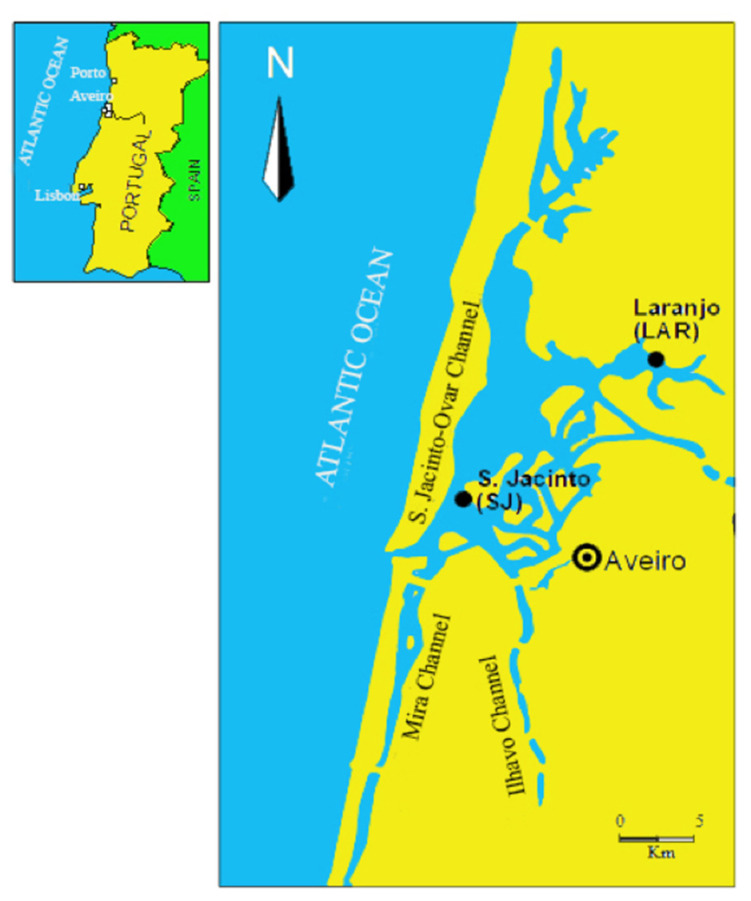
Location of the fish sampling sites at Aveiro lagoon (Portugal): São Jacinto (SJ) (40°41′00″ N, 8°42′44″ W) and Laranjo (LAR) (40°43′28.98″ N, 8°37′35.80″ W).

The Aveiro lagoon (47 km^2^ of maximum surface area) is a coastal ecosystem on the northwest coast of Portugal (Figure 1). In its upper part, there is an inner and enclosed basin named Laranjo (LAR, with around 2 km^2^) that had received Hg effluents from a chloro-alkali plant for about five decades. Although the plant activities had ceased in 1994, high levels of Hg are still stored in sediments [11] and could be found in the biota [11,17,18]. Moreover, Hg biological effects have been reported in fish [2,3,10,11,17]. Laranjo basin is a “field laboratory” for Hg toxicity investigative studies since other relevant contaminant sources besides Hg are considered negligible [19], and, therefore, it offers a unique opportunity to evaluate Hg toxicity under realistic conditions [10,11,17].

São Jacinto (SJ) is located near the lagoon entrance, distancing about 10 km from the Laranjo basin. In studies evaluating the effects of Hg in fish, SJ has been selected as a reference for comparison purposes with LAR, since it was considered unpolluted for Hg [17,18]. In fact, as already reported in our previously published papers [2,3,6], LAR presented higher levels of total dissolved Hg (1.5 ± 0.77 ng/L) and MeHg (0.040 ± 0.008 ng/L) in water than SJ (1.0 ± 0.02 ng/L and 0.016 ± 0.007 ng/L, respectively). The same spatial pattern was also recorded in the surface sediments for the total Hg (0.44 ± 0.25 µg/g at LAR and 0.025 ± 0.005 µg/g at SJ) and MeHg (0.008 ± 0.003 µg/g at LAR and 0.0001 ± 0.00002 µg/g at SJ). About the water parameters, similar temperatures of around 18 °C were recorded at both sites, while salinity was lower at LAR (average of 21) than SJ (33), thus reflecting the proximity of the sea (Figure 1). As previously reported, the levels for dissolved oxygen indicated undersaturation at LAR (average of 65%) whereas at SJ it was around 100% saturation [2,3,6].

### 2.2. Fish Sampling

Specimens of the golden grey mullet (*Chelon auratus*) were captured in summer (June 2013) at two sites of the Aveiro lagoon (Figure 1), namely SJ and LAR. A total of eight wild fish were collected at each sampling site. Sampling was carried out during low-tide using a traditional beach-seine net named “chincha”. Juvenile specimens, being not sexually mature, were selected to minimize the interference of gender on metabolome data, and thus in results interpretation. Fish total length at LAR and SJ was 13.6 ± 2.1 and 16.5 ± 2.1 cm, respectively. Immediately after catching, the fish were anesthetized, sacrificed, and properly bled in compliance with the ethical guidelines of the European Union Council (Directive 2010/63/EU), and then the gills and liver were removed. The tissue/organ samples were immediately flash-frozen in liquid nitrogen in the field, while at the lab the samples were stored at −80 °C until further processing for metabolite determinations. As previously reported, the levels of tHg that were accumulated in the gills of mullets from SJ and LAR were 0.08 and 0.22 µg/g dry weight (d.w.), respectively, whereas the concentrations of tHg in the fish liver from SJ and LAR were 0.9 and 2.3 µg/g d.w., respectively [2,3]. Moreover, the level of bioaccumulation of MeHg in the fish gills from SJ and LAR was 0.02 and 0.1 µg/g d.w., respectively, whereas in liver from SJ and LAR it was 0.6 and 1.7 µg/g d.w., respectively [2,3].

### 2.3. Chemicals

Deuterated water (D_2_O; Armar AG, Dottingen, Switzerland) and 2,2-dimethyl-2-silapentane-5-sulfonate (DSS; Sigma-Aldrich, Milan, Italy), as well as other chemicals (Sigma-Aldrich, Italy) were acquired to conduct the metabolomics analysis.

### 2.4. ^1^H NMR-Based Metabolomics

#### 2.4.1. Gills and Liver Metabolite Extraction

To extract polar metabolites, a “two-step” methanol/chloroform/water procedure [20,21,22] was applied to the frozen fish gill and liver tissue of 150 and 100 mg weight, respectively. The tissue samples were homogenized in cold methanol (4 mL/g) and cold water (0.85 mL/g) by a TissueLyser LT bead mill (Qiagen, Hilden, Germany) for 10 min at 50 vibrations/s. Then, chloroform (4 mL/g) and water (2 mL/g) were added, and the samples were vortexed for 1 min. The samples were kept on ice for 10 min, followed by centrifugation for 5 min at 2000× *g* at 4 °C to separate suspension into three phases: the water phase at the top, the denatured proteins in the middle, and the lipid phase at the bottom. The upper methanol layer with polar metabolites (600 µL) of each sample was transferred into clean microtubes and evaporated to dryness by a centrifugal vacuum concentrator (Eppendorf 5301) to be then stored at −80 °C. Before NMR analysis, the dried polar extracts were redissolved with 600 μL of sodium phosphate buffer (0.1 M, pH 7.0, 10% D_2_O) containing 1 mM of DSS, vortexed, and then transferred into a 5 mm diameter NMR tube for NMR spectroscopy. DSS was added as the internal standard providing a chemical shift reference (δ = 0.0 ppm) for the NMR spectra, whereas D_2_O provided the deuterium lock signal for the NMR spectrometer.

#### 2.4.2. ^1^H NMR Metabolomics and Spectral Pre-Processing

The ^1^H NMR spectra of all the fish samples were acquired on a Varian-500 NMR spectrometer that was operating at 499.74 MHz at 298 K. One-dimensional (1-D) ^1^H NMR spectra were obtained using a PRESAT pulse sequence to suppress the residual water resonance and 6983 Hz spectral width with a 2.0 s relaxation delay. A total of 128 transients were collected into 16,384 data points in a 10 min acquisition time. All data sets were zero filled to 32,768 data points and exponential line-broadenings of 0.5 Hz were applied prior to Fourier transformation. To reduce the complexity of the NMR data and facilitate the pattern recognition, ^1^H NMR spectra of all the fish samples were manually adjusted for phase, baseline, and calibration (DSS at 0.0 ppm) by Chenomx Processor, a module of Chenomx NMR Suite (version 5.1; Chenomx Inc., Edmonton, AB, Canada) software. The peaks within the ^1^H NMR spectra were identified with reference to known chemical shifts and peak multiplicities using public databases such as the Human Metabolome DataBase (HMDB) [23] and the Chenomx 500-MHz library database. Metabolite quantification was performed by Chenomx NMR Suite software that uses the concentration of the internal standard DSS to determine the concentrations of individual metabolites.

All 1-D ^1^H NMR spectra were converted to a data matrix using Chenomx Profiler, which segmented each spectrum into 0.005 ppm bins between 0.5 and 9.5 ppm for the gill and liver NMR spectra, with regions from 0.60 to 0.64 and 2.87 to 2.93 ppm (DSS), and 4.66 to 5.19 ppm (water) removed from all spectra to prevent interference in subsequent multivariable analyses. To conduct comparisons between the spectra, the integrated spectral area of the remaining bins was normalized before pattern recognition analysis to the total integrated area of the spectra to eliminate the dilution or bulk mass differences among the samples due to the different weight of tissue. 

### 2.5. Statistical Analysis

The data were normalized to tissue weight and then expressed in mM as a mean ± standard deviation (S.D.). With the aim to clearly recognize the similarities and differences in the metabolome of the two examined fish organs, a Venn diagram was applied using the web application GeneVenn [24]. Moreover, to highlight any potential deviations from the metabolome profiles of the gills and liver of the fish that were triggered by environmental exposure to Hg, a principal component analysis (PCA) was conducted using MATLAB, version R2016a (The MathWorks Inc., Natick, MT, USA) to reduce the dimensionality of the metabolomic data and distinguish the two organs and the two groups of fish (from SJ and LAR). In fact, the PCA allows differences and similarities among the NMR metabolic fingerprints to be visualized in a score plot since samples that are metabolically similar are clustered together. The organ-specific metabolite changes were then calculated via the ratio between the means of LAR and SJ individual metabolites as measured within each organ. The metabolic dataset was tested for normality using the Shapiro-Wilk distribution test, and after confirmation of normal distribution, data homogeneity was evaluated through the Levene’s test. Univariate statistical analysis, namely the parametric Student’s *t*-test, was therefore carried out on metabolite data using the GraphPad software (Prism 5.0, San Diego, CA, USA). The threshold for significance was *p* < 0.05, recognized as the criterion of statistical significance.

## 3. Results

### 3.1. Metabolite Profiling of Fish Gills and Liver from the Reference Area

**Figure 2:**
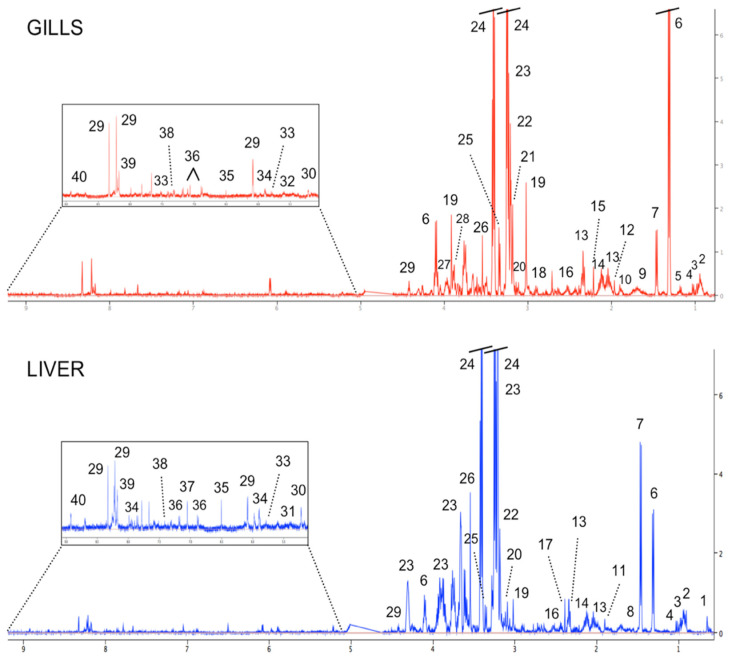
Representative 1-D 500 MHz ^1^H NMR spectra of the gills and liver of golden grey mullet (*Chelon auratus*) that were collected from SJ. Keys: (1) taurocholic acid, (2) leucine, (3) isoleucine, (4) valine, (5) isobutyrate, (6) lactate, (7) alanine, (8) unknown resonance #1, (9) lysine, (10) arginine, (11) acetate, (12) N6-acetyllysine, (13) glutamate, (14) glutamine, (15) acetone, (16) glutathione, (17) succinate, (18) aspartate, (19) creatine, (20) malonate, (21) choline, (22) phosphocholine, (23) glycerophosphocholine, (24) taurine, (25) unknown resonance #2, (26) glycine, (27) serine, (28) betaine, (29) inosine, (30) glucose, (31) glycogen, (32) UDP-glucose, (33) uracil, (34) uridine, (35) fumarate, (36) tyrosine, (37) unknown resonance #3, (38) phenylalanine, (39) hypoxanthine, and (40) niacinamide.

The representative 1-D ^1^H NMR spectra of tissue extracts of the gills and liver from golden grey mullet (*C. auratus*) that were sampled from the reference area (SJ) are depicted in Figure 2. Numerous metabolites were identified within the metabolome of both organs, which were both characterized by a dominant presence of the organic osmolyte taurine (3.25 and 3.41 ppm), found at a concentration of approximately 45 times higher than other metabolites in both the gills and liver. Moreover, all spectra of fish gills were also dominated by lactate (1.33 and 4.12 ppm), which was about 20 times higher than other metabolites, whereas within the ^1^H NMR spectra of the fish liver the second dominant metabolite was glycerophosphocholine that was found to be approximately 15 times more concentrated than other metabolites. Other major classes of compounds that were found in the metabolome of both fish organs included amino acids (e.g., glutamate, alanine), energy storage compounds (e.g., glucose), glycolytic products (e.g., lactate), Kreb’s cycle intermediates (e.g., malonate, fumarate in gills; succinate in liver), and nucleotides and their derivatives (e.g., uracil).

A list of the metabolites that were identified, with their relative chemical shifts and concentrations, is reported in Table 1.

### 3.2. Metabolome Comparison between Fish Gills and Liver from the Reference Area

**Figure 3:**
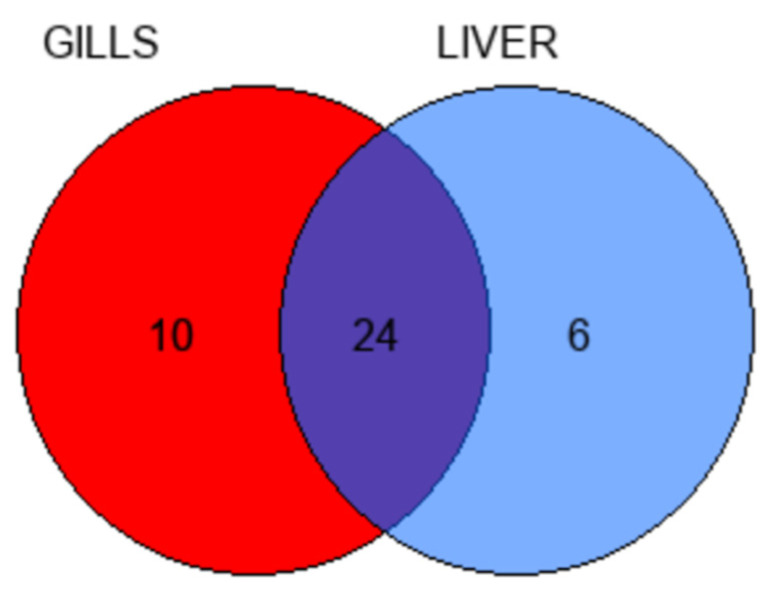
Venn diagram showing the overlap of the metabolites that were detected in the gills and liver of fish that were collected from the reference area.

To evaluate the similarities and differences in the metabolome content of the two organs under investigation, all the metabolites that were identified in the gills and livers of the fish from the reference area were compared by performing a Venn diagram, as shown in Figure 3. From the comparison of fish gills and liver metabolomes, a total of 40 polar metabolites that are involved in a variety of metabolic pathways were found in the two examined organs. In detail, 24 out of these 40 metabolites were commonly detected in both the fish gills and liver (Figure 3 and Table 2), even if the concentrations of most of these metabolites differed between the two tissues. Moreover, the Venn diagram revealed that ten metabolites were found solely in the fish gills (i.e., acetone, arginine, aspartate, betaine, choline, isobutyrate, lysine, N6-acetyllysine, serine, and UDP-glucose), while six metabolites were detected exclusively in the fish liver (i.e., acetate, glycogen, succinate, taurocholic acid, unknown resonance #1, and unknown resonance #3), as reported in Figure 3 and Table 2. It is worthy to note that the metabolites that were found exclusively in an organ may have been not detected in the other because of their low concentration with respect to the sensitivity of NMR spectroscopy.

### 3.3. Metabolome Changes Induced by Hg in Fish Gills and Liver

**Figure 4:**
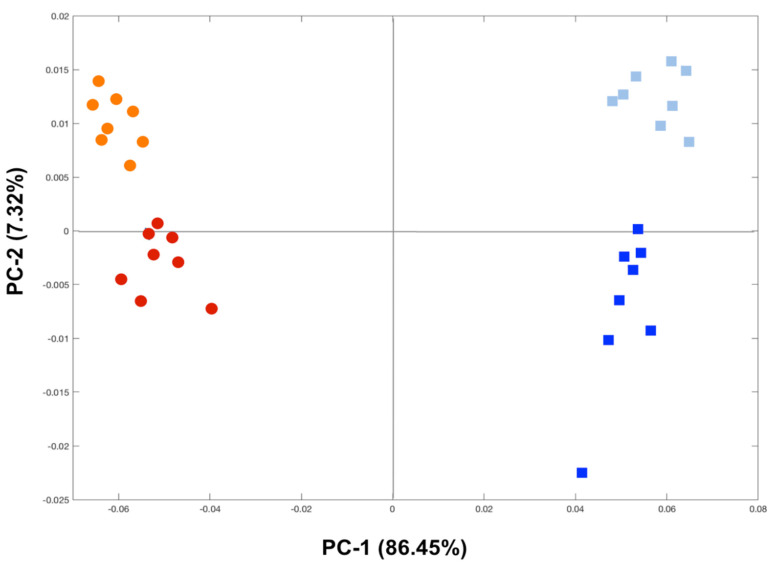
Principal component analysis (PCA) of ^1^H NMR spectra of golden grey mullet gills (circles) and liver (squares) showing separation (PC-1 vs. PC-2) between the fish that were collected at SJ (orange circles and light blue squares) and those from LAR (red circles and blue squares).

The PCA scores plot of the ^1^H NMR metabolic profiles confirmed the metabolome differences between the fish gills (circles) and the liver (squares), as evidenced along the PC-1 axis and explained by 86.45% of variance (Figure 4). Moreover, a clear clustering of the fish groups from the two sampling sites was also noticed for both tissues along the PC-2 axis that, with the 7.32% of variance, separated mullets that were sampled from SJ (orange circles and light blue squares) from those that were collected at LAR (red circles and blue squares) (Figure 4). The metabolites that were responsible for the differences that were observed between the two groups of fish for each tissue under examination were identified as the result of statistical analysis. As reported in Table 3, the metabolic gill fingerprints of the wild fish that were sampled from LAR compared to those from SJ were characterized by significantly higher levels of alanine, creatine, lactate, uracil, and choline, together with significantly reduced levels of taurine, glycerophosphocholine, and glutathione. Conversely, the metabolic profiles of the livers of mullets that were collected at LAR in respect to those from SJ evidenced significantly elevated concentrations of alanine, phosphocholine, glucose, and glutathione, besides significantly decreased levels of tyrosine, phenylalanine, taurine, and hypoxanthine, as reported in Table 3.

## 4. Discussion

### 4.1. Metabolome of Fish Gills and Liver from the Reference Area

Metabolomics is a powerful approach in elucidating the interactions between organisms and the environment [4,5]. However, the necessity to correctly evaluate the metabolome of the biological system under investigation to accurately address the toxicity of aquatic contaminants avoiding interferences of confounding factors is undeniable. Therefore, a ^1^H NMR-based metabolomic approach was herein conducted on the gills and liver, the target organs that are strongly connected to each other in terms of metabolism, of the wild golden grey mullet (*Chelon auratus*) that were collected from a reference area explore and compare their metabolome to highlight any qualitative and/or quantitative differences or similarities in their metabolites, to be attributed to their major functions. Specifically, the gills of fish are mainly involved into the respiratory gas exchange activity, active ion transport [25], osmoregulation [26], acid-base balance, and excretion of nitrogenous wastes [27], besides being the main route for uptake of waterborne environmental pollutants. Contrarily, the liver of fish is a target organ for a number of metabolic activities and detoxification processes, besides playing a main role in accumulation, biotransformation, and cycling of environmental pollutants, including Hg [28,29,30,31].

From a careful comparative evaluation of the ^1^H NMR spectra of the gills and liver of mullets from the reference area, a total of 40 metabolites were identified and, according to their metabolic roles, clustered into six groups including the metabolites that are involved in the metabolism of amino acids, comprising of 16 compounds, in the energy metabolism (8 compounds), in the osmoregulatory processes (3 compounds), in nucleotide and their derivative metabolism (4 compounds), in bile acid metabolism (1 compound), and miscellaneous metabolic pathways (5 compounds). Additionally, three unknown resonances were found in the fish liver, with only the unknown resonance #2 commonly present also in the gills but at a concentration that was notably higher than that which was recorded in the liver. This further supports the hypothesis of an organ-specific variation, both qualitative and quantitative, in metabolites between the two fish organs that were examined that is strictly correlated to physiological specificities. Interestingly, 60% of the identified metabolites were commonly present in fish gills and liver, although exhibiting different concentrations.

It is worthy to note that the metabolome of both organs was found to be dominated by the organic osmolyte taurine, detected at a concentration of about 45 times higher than that of other metabolites. Taurine is a sulfur-containing amino acid that serves primarily in the maintenance of cellular homeostasis. For instance, in case of hypo-osmotic stress, the induced cell swelling is followed by a regulatory volume decrease due to cellular efflux of ions and organic osmolytes, including taurine [32]. It is, therefore, justified that the high level of taurine that was recorded in the metabolome of fish gills from the reference area that, being in direct contact with the surrounding water, constantly deal with fluctuations of water salinity. A high sensitiveness to environmental osmolarity is peculiar of euryhaline fish, such as golden grey mullets and European sea bass (*Dicentrarchus labrax*), as also demonstrated by in vitro investigations [33]. However, for the liver that is not directly challenged by the external medium, the dominant presence of taurine may be explained by its well-documented metal-chelating and antioxidant properties, and, therefore, its active involvement in detoxifying processes and hepatoprotective effects [34].

Among organic osmolytes, besides betaine that was detected exclusively in the fish gills as further evidence of the osmoregulatory processes that actively occur within fish branchial epithelium, it is important to highlight the common presence in the two fish organs of glycerophosphocholine, found at a notably higher level in the liver than the gills metabolome. Indeed, besides its role into the mechanisms of osmoregulation, it must be noted that glycerophosphocholine, together with phosphocholine, are the main storage forms for choline within the cytosol [35] and, as a matter of fact, no choline was found in the liver of golden grey mullets.

As stated above, the largest group of metabolites that was identified was that of the amino acids, even if it must be pointed out that, contrarily to gill metabolome, in the liver not all amino acids were detected. This discrepancy is likely to be associated to the different role of the examined organs. In detail, it is evident that there is a higher level of alanine in the liver compared to the gill metabolome, and this may be attributed to its role as main carrier of amino nitrogen to liver. However, the presence of alanine in the gills, although at a lesser extent than in the liver, may be associated with the nitrogenous waste excretion that is occurring at the branchial epithelium [27]. In regard to the branched-chain amino acids (BCAAs), namely leucine, isoleucine, and valine, they were all found at comparatively similar concentrations in both organs, although at a slightly higher extent in the liver than the gills. BCAAs are known essential amino acids for the immune system because, beyond their role in the regulation of protein turnover processes, they serve as proteinogenic amino acids, precursors for the biosynthesis of new molecules and cells, such as lymphocytes [36], but also as energy sources. 

Among the differences in amino acids content between the fish gills and liver, isobutyrate, lysine, arginine, aspartate, N6-acetyllysine, and serine were observed only in the gills. Among these, isobutyrate is recognized as a marker of anoxia, of which the concentration increases in the gills, is involved in the respiratory gas exchange activity when the organism is facing a hypoxic condition due to alteration in the oxygen intake [37,38]. The absence of isobutyrate in the metabolome of the fish liver is, therefore, not a surprise, due to the different physiological role in respect to gills.

Metabolites that are involved in the energy metabolism were also found in both the fish gills and liver metabolome, though with evident differences between the organs in terms of the metabolites that were detected or in their concentrations. In detail, the fish liver was characterized by higher levels of glucose and glycogen, the latter of which was not recorded in the gills, and higher concentrations of Kreb’s cycle intermediates together with a lower level of lactate. This metabolite profiling of the liver finds explanation considering that, among the others, the liver plays a unique role in controlling the carbohydrate metabolism and lipid homeostasis [39].

Another class of compounds that was found in the metabolite profiling of fish gills and liver was that of nucleotides and their derivatives, including uracil, uridine, hypoxanthine, and inosine, where the latter was found to be the only molecule where the concentration was higher in the gills than the liver. Overall, the different levels of these compounds as found between fish organs may reflect ongoing transcriptional activities. Thus, it is reasonable to hypothesize that the concentrations of nucleotides and their derivatives are highly variable, context-specific, and strictly associated with tissue-specific metabolism [40].

Furthermore, a metabolite that was recorded in fish liver and not detected in the gills was the taurocholic acid, a bile acid representing the product of conjugation of cholic acid with taurine. Bile acids, known as essential organic molecules that are synthesized in the liver from cholesterol, are also recognized as valid indicators of hepatobiliary impairment because their synthesis and metabolism are strictly influenced by liver diseases [41].

### 4.2. Tissue-Specific Metabolic Changes Induced by Hg in Fish Gills and Liver

Data on Hg accumulation in golden grey mullets that were collected at LAR and compared to SJ, as reported above and published previously [2,3], revealed a bioaccumulation of tHg and MeHg level of 10- and 17-fold higher, respectively, in fish liver compared to the gills, thus making it feasible to hypothesize the induction of metabolic changes in both fish organs. Therefore, a further purpose of this study was to elucidate similar or differential deviations from the metabolome profiles of the gills and liver of mullets that were triggered by environmental exposures to Hg. However, besides Hg, the influence on some key metabolites of the differential salinity and dissolved oxygen as recorded at the two sampling sites was also considered in this field study.

A clear grouping was observed between the two fish organs that were examined, as well as between the two groups of fish that were collected at SJ and LAR, thus suggesting a clear differentiation in their metabolic profile. From the comparison of the major metabolites that were probably affected by the exposure to Hg, it was possible to notice a different extent of perturbation or an opposite or specific pattern in the changes of metabolites between the fish gills and liver. This reflects differential toxicological effects that were triggered by Hg exposure in each organ, as well as their ability to respond to the same stressor by activating organ-specific adaptive and defensive strategies.

Of note, a significant reduction in taurine level that was induced by Hg exposure was observed in both the fish gills and liver, even if likely to be due to different Hg modes of action. In fact, in the gills it may be attributed to disturbances in osmoregulatory processes since taurine is known to be involved in the maintenance of cellular homeostasis [32,33]. Therefore, the decreased taurine level that was recorded in the gills from LAR with respect to those from SJ may reflect interferences in osmotic balance due to differences in salinity between the two sampling sites as well as to Hg bioaccumulated levels. Conversely, in the liver, that is an internal organ and thus not markedly challenged by the external medium, the influence of salinity in taurine level could be disregarded. Its depletion may be explained with the taurine metal-chelating property, and, therefore, its active involvement in detoxifying and hepatoprotective effects [34]. Moreover, taurine can act as a potent antioxidant and its reduced levels that were observed in both fish organs may thus be indicative of a protective mechanism against Hg-induced oxidative stress [42], which was demonstrated to occur in both the mullet gills and liver in our previous studies through oxidative stress endpoints [2,3,6]. It is also plausible to hypothesize that if taurine is affording antioxidant protection, this could compromise other taurine-dependent functions.

A similar trend in the metabolic change that was induced by Hg in fish gills and liver was also found for alanine, which increased in both tissues after Hg exposure. Taking into consideration that alanine is the main nitrogen carrier to the liver, its augmentation in both organs may be explained as an adaptive response to deal with an excess of free ammonia, maybe resulting from protein catabolism to prevent intoxication in gills, which is actively involved also in nitrogenous waste elimination [27].

Surprisingly, opposite changes, even if not all significant, were observed in mostly of the Hg-altered metabolites between the fish gills and liver. This is the case of glycerophosphocholine and phosphocholine, which depleted both in the gills, showing an opposite trend than choline, but both increased in the liver. The significant decrease of glycerophosphocholine that was measured in the gills is related to changes in osmoregulatory processes and, therefore, is affected, not only by Hg but also by changes in salinity as found between the two sampling sites. Moreover, it may be linked to ongoing membrane stabilization/repair processes due to the biosynthesis of phosphatidylcholine, the major structural phospholipid of cell membranes [6], as an adaptive cell-protecting antioxidant mechanism that is occurring in mullet gills from LAR to cope with decreased dissolved oxygen and environmental Hg contamination [3]. Contrarily, the opposite trend that was exhibited by the same metabolites in the fish liver suggests the breakdown of phosphatidylcholine and/or the occurrence of membrane turnover. The increase in phosphatidylcholine degradation products suggest the occurrence of oxidative insult at the hepatic level, more likely triggered by Hg exposure as specifically documented in our previous paper [2]. Interestingly, aberrant choline metabolism is a hallmark of oncogenesis and cancer progression that is characterized by increased phosphocholine, glycerophosphocholine, and total-choline-containing compounds that are also strictly related to the Kennedy pathway, which constitutes the biosynthesis pathway for membrane phosphatidylcholine [43].

An opposite trend between the two organs was also noticed for glutathione, which significant dropped in the fish gills together with the reduced levels of its constituent amino acids (i.e., glycine and glutamate). This makes it feasible to hypothesize some vulnerability in mullet gills to Hg toxicity, as the reduction in glutathione enhances the risk of oxidative stress due to the accumulation of reactive oxidative species that is triggered by environmental Hg pollution as well as related to the decreased dissolved oxygen [6,44]. Interestingly, an accurate evaluation of the pro-oxidant status of mullet gills from LAR indicated the occurrence of massive GSH oxidation under Hg stress and an inability to carry out its regeneration or de novo synthesis [3]. On the contrary, the significant hepatic elevation in glutathione content suggests an adaptive response to Hg bioaccumulation [31], resulting in the activation of antioxidant defense mechanisms in mullets that are exposed to Hg, further supporting the organ-specific functions with liver being the major site of storage and detoxification of pollutants.

Among metabolites exhibiting opposite patterns between the two fish organs, lactate must also be mentioned. Its significant increase in the gills of Hg-exposed mullets may be indicative of an enhanced anaerobic metabolism as an adaptive strategy to replenish insufficient energy supply due to environmental Hg contamination as well as to the lower oxygen availability that was recorded at LAR [6]. On the contrary, the observed hepatic dropping of lactate may be related to its use as a substrate for gluconeogenesis, as further supported by the elevated levels of glucose in the liver together with unaltered glycogen levels [31]. Therefore, this may be considered as a further organ-specific toxicological effect that is induced by Hg in fish.

It is worthy to also note the significant rise in the level of creatine in the gills of fish that were exposed to Hg, which indicates changes in ion-osmoregulatory processes, and therefore, may also be influenced by the lower salinity that was recorded at LAR. In regard to the hepatic creatine level, no changes were detected in the fish from the Hg-contaminated area, LAR. However, considering that the liver is usually the site of creatine production, which has to be synthesized continuously [45], it is reasonable to hypothesize that as creatine is biosynthesized it is soon transported to the blood to be translocated to the gills [46] to cope with Hg-induced changes in ion-osmotic balance.

## 5. Conclusions

Proton NMR-based metabolomics allowed a successful comparison of the complete metabolome of the gills and liver of golden grey mullets (*C. auratus*), revealing similarities and differences in their metabolite profile to be related to their organ-specific roles. Among others, taurine was predominant in both organs and is mainly involved in osmoregulation in the gills and detoxification and antioxidant protective processes in the liver. Also, the high levels of glucose and glycogen that were observed in the liver confirmed its role in carbohydrate storage.

Mercury environmental contamination triggered multiple and often differential metabolic alterations in the golden grey mullet gills and liver. In detail, severe disturbances in ion-osmoregulatory processes were highlighted in the gills, whereas differential impairments in energy-producing metabolic pathways and protein catabolism were found between the fish organs. Perturbation in membrane stabilization processes and alteration of the antioxidant defense system were also pointed out, thus reflecting differential cytotoxicological effects that were triggered by environmental Hg, as well as their ability to respond to the same stressor by activating organ-specific adaptive and defensive strategies.

Overall, a strict correlation between metabolites and the organ-specific physiology of the gills and liver were discerned, which provides precious information for a better understanding of metabolite shifts when investigating the cytotoxicity of Hg in fish.

## Figures and Tables

**Table 1:** ^1^H NMR measurements (mM) as means ± SD of metabolites that were found in the gills and liver of golden grey mullet (*C. auratus*) that were collected from the reference area (s: singlet; d: doublet; t: triplet; q: quartet; dd: doublet of doublets; dt: doublet of triplets; dq: doublet of quartets; m: multiplet).

Metabolites Involved to:	Chemical Shift and Peak Shape, ppm	GILLS	LIVER
**Amino Acid Metabolism**			
Isoleucine	0.92 (t), 1.00 (d), 1.26 (m), 1.44 (m), 1.96 (m), 3.66 (d)	0.33 ± 0.03	0.69 ± 0.15
Leucine	0.94 (d), 0.96 (d), 1.66 (m), 3.71 (t)	0.87 ± 0.09	1.11 ± 0.24
Valine	0.98 (d), 1.03 (d), 2.25 (m), 3.59 (d)	0.62 ± 0.06	0.92 ± 0.18
Isobutyrate	1.19 (d), 2.59 (m)	0.51 ± 0.11	-
Alanine	1.46 (d), 3.76 (m)	4.28 ± 0.46	11.25 ± 2.88
Arginine	1.68 (m), 1.90 (m), 3.23 (t), 3.74 (t)	1.64 ± 0.22	-
Lysine	1.48 (m), 1.73 (m), 1.91 (m), 3.03 (t), 3.76 (t)	0.58 ± 0.13	-
N6-acetyllysine	1.41 (m), 1.56 (m), 1.87 (m), 1.98 (s), 3.19 (q), 3.74 (t)	0.46 ± 0.04	-
Glutamate	2.08 (m), 2.34 (m), 3.74 (t)	7.61 ± 0.93	10.76 ± 2.23
Glutamine	2.12 (m), 2.44 (m), 3.75 (t)	1.16 ± 0.16	2.17 ± 0.32
Aspartate	2.66 (dd), 2.79 (dd), 3.87 (dd)	1.15 ± 0.17	-
Creatine	3.02 (s), 3.91 (s)	2.16 ± 0.62	1.27 ± 0.13
Serine	3.84 (dd), 3.95 (m)	2.76 ± 0.19	-
Glycine	3.54 (s)	3.23 ± 0.19	6.66 ± 1.56
Tyrosine	6.89 (d), 7.19 (d)	0.48 ± 0.08	0.87 ± 0.13
Phenylalanine	3.13 (m), 3.28 (m), 3.98 (m), 7.31 (d), 7.36 (t), 7.41 (m)	0.49 ± 0.07	0.99 ± 0.12
**Energy metabolism**			
Acetate	1.91 (s)	-	0.67 ± 0.12
Succinate	2.41 (s)	-	0.95 ± 0.16
Malonate	3.13 (s)	0.79 ± 0.07	1.62 ± 0.41
Glucose	3.23 (m), 3.40 (m), 3.45 (m), 3.52 (dd), 3.73 (m), 3.82 (m), 3.88 (dd), 4.63 (d), 5.22 (d)	2.01 ± 0.25	6.87 ± 1.51
Glycogen	3.40 (m), 3.60 (m), 3.80 (m), 3.96 (s), 5.40 (s)	-	1.35 ± 0.27
Lactate	1.33 (d), 4.12 (q)	19.84 ± 3.82	10.09 ± 1.68
Fumarate	6.51 (s)	0.17 ± 0.01	0.54 ± 0.06
UDP-glucose	3.49 (m), 3.72 (d), 4.12 (m), 4.27 (m), 4.37 (m), 5.63 (q), 5.96 (d), 6.71 (d), 8.11 (d)	0.36 ± 0.04	-
**Osmoregulation**			
Betaine	3.25 (s), 3.89 (s)	1.04 ± 0.21	-
Taurine	3.25 (s), 3.41 (t)	45.84 ± 6.12	41.83 ± 11.21
Glycerophosphocholine	3.21 (s), 3.60 (dd), 3.67 (m), 3.90 (m), 4.31 (m)	1.96 ± 0.53	14.54 ± 2.61
**Nucleotide and their derivative metabolism**			
Uracil	5.81 (d), 7.54 (d)	0.47 ± 0.05	1.28 ± 0.36
Uridine	3.8 (dd), 3.9 (dd), 4.1 (q), 4.2 (t), 4.3 (t), 5.9 (dd), 7.9 (d)	0.45 ± 0.06	1.22 ± 0.29
Hypoxanthine	8.17 (s), 8.20 (s)	1.07 ± 0.15	1.42 ± 0.25
Inosine	3.83 (dd), 3.90 (dd), 4.27 (dd), 4.25 (t), 4.76 (t), 6.08 (d), 8.21 (s), 8.33 (s)	3.02 ± 0.27	1.83 ± 0.31
**Bile acid metabolism**			
Taurocholic acid	0.65 (s), 0.91 (s), 0.98 (m), 1.36 (m), 1.42 (m), 1.61 (m), 1.96 (m), 2.13 (d), 2.20 (m), 2.53 (t), 3.18 (m), 3.28 (dd), 3.61 (s), 3.78 (s)	-	1.59 ± 0.76
**Miscellaneous metabolic pathways**			
Choline	3.21 (s), 3.52 (s), 4.07 (m)	0.89 ± 0.15	-
Glutathione	2.13 (m), 2.54 (m), 2.97 (dd), 3.75 (m), 4.53 (m)	1.17 ± 0.23	1.72 ± 0.22
Niacinamide	7.58 (dq), 8.24 (dt), 8.70 (dd), 8.92 (d)	0.52 ± 0.04	2.33 ± 0.42
Acetone	2.22 (s)	0.83 ± 0.22	-
Phosphocholine	3.21 (s), 3.57 (t), 4.16 (m)	1.06 ± 0.15	0.95 ± 0.38
**Unknown resonances**			
Unknown resonance #1	1.52 (m)	-	1.82 ± 0.68
Unknown resonance #2	3.36 (d)	2.88 ± 0.36	0.76 ± 0.11
Unknown resonance #3	7.05 (s)	-	2.55 ± 0.58

**Table 2:** Metabolite comparison between the gills and liver of golden grey mullet (*C. auratus*) that were sampled from the reference area São Jacinto (SJ), northwest coast of Portugal.

Description	Metabolites
Both in fish gills and liver	Leucine; Isoleucine; Valine; Lactate; Alanine; Glutamate; Glutamine; Glutathione; Creatine; Malonate; Phosphocholine; Glycerophosphocholine; Taurine; Glycine; Inosine; Glucose; Uracil; Uridine; Fumarate; Tyrosine; Phenylalanine; Niacinamide; Hypoxanthine; Unknown resonance #2
Only in fish gills	Acetone; Arginine; Aspartate; Betaine; Choline; Isobutyrate; Lysine; N6-acetyllysine; Serine; UDP-glucose
Only in fish liver	Acetate; Glycogen; Succinate; Taurocholic acid; Unknown resonance #1; Unknown resonance #3

**Table 3:** The relative changes in metabolite concentrations between LAR and SJ golden grey mullets (*p* < 0.05 ^a^; Student’s *t* test) in both the gills and liver (+ and − indicate metabolite increase and decrease, respectively, in the organs of fish from LAR with respect to those from SJ; “n.c.” indicates no changes that were induced by Hg exposure).

Metabolites Involved to:	GILLS	LIVER
**Amino Acid Metabolism**		
Isoleucine	+34%	−15%
Leucine	+27%	−21%
Valine	+21%	−17%
Isobutyrate	+42%	Not found
Alanine	+24% ^a^	+37% ^a^
Glutamate	−22%	+24%
Creatine	+43% ^a^	n.c.
Glycine	−26%	n.c.
Serine	−18%	Not found
Tyrosine	n.c.	−28% ^a^
Phenylalanine	n.c.	−25% ^a^
**Energy metabolism**		
Lactate	+38% ^a^	−44%
Fumarate	+35%	n.c.
Succinate	Not found	+12%
Glucose	n.c.	+52% ^a^
Glycogen	Not found	n.c.
**Osmoregulation**		
Taurine	−52% ^a^	−38% ^a^
Glycerophosphocholine	−32% ^a^	+23%
**Nucleoside and their derivative metabolism**		
Inosine	−18%	−16%
Uracil	+25% ^a^	−32%
Hypoxanthine	n.c.	−45% ^a^
**Miscellaneous metabolic pathways**		
Glutathione	−38% ^a^	+56% ^a^
Choline	+48% ^a^	Not found
Phosphocholine	−19%	+93% ^a^

## Data Availability

The data presented in this study are available on request from the corresponding author.
